# Foundational insights for theranostic applications of magnetoelectric nanoparticles

**DOI:** 10.1039/d4nh00560k

**Published:** 2025-01-23

**Authors:** Victoria Andre, Mostafa Abdel-Mottaleb, Max Shotbolt, Shawnus Chen, Zeinab Ramezini, Elric Zhang, Skye Conlan, Ozzie Telisman, Ping Liang, John M. Bryant, Roman Chomko, Sakhrat Khizroev

**Affiliations:** a Department of Biomedical Engineering, University of Miami Coral Gables FL USA; b Department of Chemical, Environmental and Materials Engineering, University of Miami Coral Gables FL USA; c Department of Electrical and Computer Engineering, University of Miami Coral Gables FL USA skhizroev@miami.edu; d Department of Chemistry, University of Miami Coral Gables FL USA; e Cellular Nanomed Irvine CA USA; f H. Lee Moffitt Cancer Center and Research Institute Tampa Florida USA; g Department of Electrical and Computer Engineering, University of California Riverside CA USA; h The Miami Project to Cure Paralysis, Department of Biochemistry and Molecular Biology, University of Miami Miami FL USA

## Abstract

Reviewing emerging biomedical applications of MagnetoElectric NanoParticles (MENPs), this paper presents basic physics considerations to help understand the possibility of future theranostic applications. Currently emerging applications include wireless non-surgical neural modulation and recording, functional brain mapping, high-specificity cell electroporation for targeted cancer therapies, targeted drug delivery, early screening and diagnostics, and others. Using an *ab initio* analysis, each application is discussed from the perspective of its fundamental limitations. Furthermore, the review identifies the most eminent challenges and offers potential engineering solutions on the pathway to implement each application and combine the therapeutic and diagnostic capabilities of the nanoparticles.

## Abstract 2

An overview of the MENP biological applications discussed in this paper, which have the potential to form theranostic systems in the treatment of various diseases.

## Graphical abstract

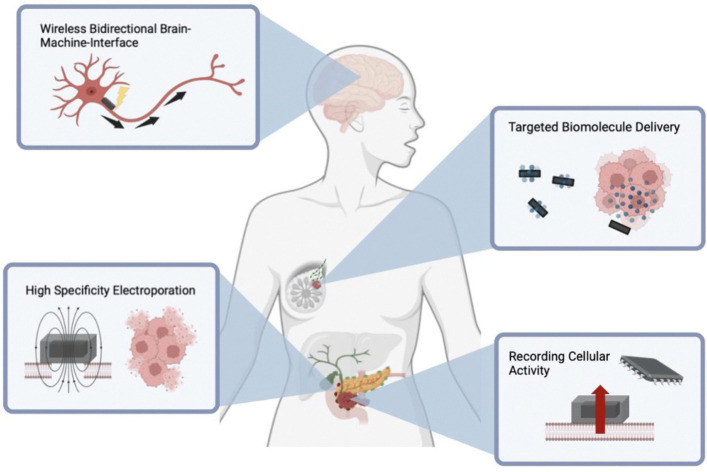

## Introduction

Theranostic techniques allow for the simultaneous diagnoses and treatment of diseases, thus paving the way for precision medicine.^1,2^ In the last decade, MENPs have been proposed and investigated as a solution to achieve wireless non-surgical control of local electric fields in biomedical systems.^1–3^ The main property that distinguishes MENPs from other nanoparticles is their magnetoelectricity, which is quantified through the magnetoelectric (ME) effect.^4–7^ As described below in more detail, magnetoelectricity provides a two-way wireless interface between a biological system, *e.g.*, the brain, and a computer, without the need for genetic modification or surgical interference. Owing to the ME effect, MENPs enable both electric-to-magnetic and magnetic-to-electric local field transformations, with the locality scale defined by the characteristic nanoparticle size and ideally providing a molecular level resolution. As a result, MENPs are naturally suited for theranostic applications.

To date, for most biomedical applications of MENPs, this theranostic interface could be achieved with core–shell MENPs.^8,9^ The two-phase core–shell MENP system has an orders of magnitude higher ME coefficient compared to that of any single-phase multiferroic system.^10–12^ The key physical properties of both core–shell and multiferroic MENPs, have been studied in detail and described in recent review papers.^12^ This paper does not focus on the materials’ properties of the nanoparticles *per se* but rather on their properties related to the field-controlled interaction with biological microenvironments. Upon application of a magnetic field, a strain propagates through the interface from the core to the shell, leading to generation of a dipole electric moment in the shell, thus inducing a local electric field in the nanoparticle's vicinity. This effect, known as the direct ME effect is the driving force of the MENPs’ therapeutic applications. For example, it can be used for local wirelessly controlled cellular modulation, *e.g.*, to induce local neural activity deep in the brain, for peripheral nerve stimulation,^13^ targeted drug delivery and release on demand, neurogenesis, trigger wireless irreversible electroporation (IRE) for high-specificity cancer therapy, or another therapeutic function.^14–17^ Conversely, upon application of a local electric field, a strain propagates through the interface from the shell to the core, thus leading to a magnetization change that can be detected remotely using a magnetic field sensor (magnetometer).^18^ This effect, known as the converse ME effect, is the driving force of the MENPs’ diagnostic applications. For example, the converse effect could be used for wireless recording of any local electric field change due to cellular activity, whether it is due to neural firing, a difference in the dielectric properties between different cell types, or another function; in turn, the resulting nanoparticles’ magnetic moment change can be detected *via* functionalized magnetic resonance imaging (MRI), magnetic particle imaging (MPI) response, or another magnetometer-driven imaging.^19–25^

**Fig. 1:**
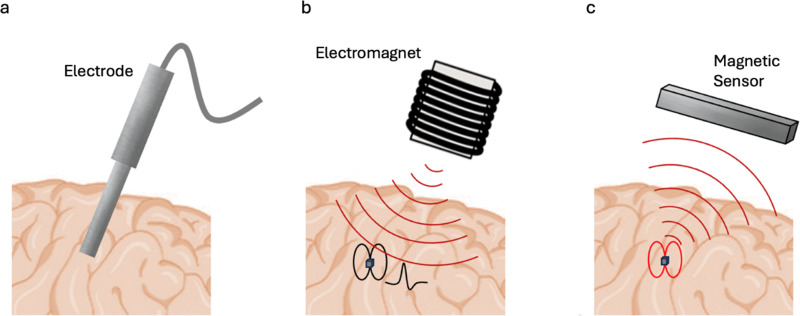
Comparison of electrode-based stimulation and recording with MENP-based stimulation and recording. (a) Electrode-based stimulation and recording in the brain. (b) Therapeutic procedure of brain stimulation using MENP-based system and an external electromagnet. (c) Diagnostic procedure of neural recording using MENP-based system and an external magnetic sensor. The illustrations are not to scale.

To underscore the significance of these magnetic-to-electric and electric-to-magnetic field transformations, vital for therapeutic and diagnostic functions, respectively, it can be reminded that electric fields alone cannot be used for wireless modulation or detection of cellular activity deep in the brain, or any other biological tissue, as these tissues consist mostly of conductive media,^26,27^ implying that any locally induced electric field would be screened out by free ions in the media with the Debye length in the sub-1-nm size range.^26^ In contrast, owing to the ME effect, MENPs can both generate and detect local electric fields even in a conductive microenvironment, thus eliminating the need for physical microelectrodes in both modulation and recording (Fig. 1).^28^ In this case, magnetic fields which, unlike electric fields, can penetrate both conducting and dielectric tissues, with no visible dissipation, serve as a wireless link replacing the physical wires in traditional microelectrode-based implants.

Given the fundamental nature of this wireless two-way control, ideally at the molecular level, one can envision many potentially groundbreaking biomedical applications of MENPs. A few examples would include (i) two-way wireless brain-machine interface (BMI) for non-medical and medical purposes, (ii) wirelessly controlled highly targeted cellular reversible and irreversible nano-electroporation for treatment of untreatable cancers, (iii) targeted drug delivery, (iv) stimulation of neurogenesis, and others. Recently, many theoretical studies as well as *in vitro*, *ex vivo* and *in vivo* experiments to prove the feasibility of these applications have been reported.^20,28–43^

However, despite the potentially game-changing biomedical applications of MENPs, major development remains to be carried out before these nanoparticles can become available in the clinic. This article points out current implementation challenges related to the physics that underlies the field-controlled interaction between MENPs and cellular microenvironment. It also offers potential solutions to the challenges based on insights learned from the over-a-decade-long research in this laboratory as well as from the recent surge of independent studies in other laboratories.^2,9^

## Physics of nanoparticle–cellular-microenvironment interaction

As mentioned above, the ME effect is the main property that distinguishes MENPs from other nanoparticles and represents the main mechanism for theranostic applications. The phenomenon of magnetoelectricity is what provides the energy conversion (from magnetic to electric field and *vice versa*) required for wirelessly connecting to the fundamental electric circuitry of the human body, with externally controlled magnetic fields substituting physical electrodes used in conventional approaches.^12^ Besides MENPs, there are other ways to achieve the required magnetoelectricity, for example, by using the electromotive force (emf) or just relatively large size magnetoelectric implants.^32^ However, it is the MENPs which can produce the effect of magnetoelectricity with the nanoscale resolution, thus ideally allowing a molecular level control of fundamental biological processes.

**Fig. 2:**
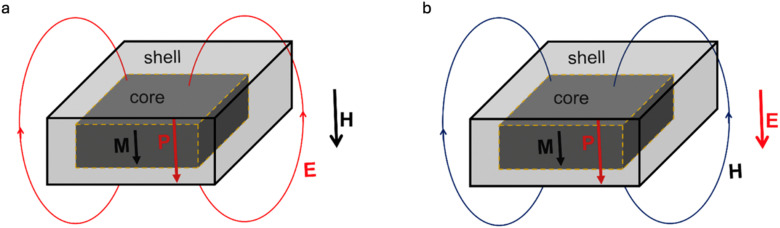
ME effect of MENPs – the driver of theranostic applications. (a) Most therapeutic approaches with MENPs are governed by application of a magnetic field to induce a local electric field in the nanoparticle's vicinity, with the field coupling described *via* the direct ME coefficient. (b) Most diagnostic approaches with MENPs are through generation of a magnetic field in response to a local electric field in the nanoparticle's vicinity, with the field coupling described *via* the converse ME coefficient. The core and shell components are made of magnetostrictive and piezoelectric materials, respectively. The two components are lattice-matched at their surface interface.

To date, most biomedical applications of MENPs have been developed using a core–shell nanocomposite system (Fig. 2).^8,9^ The two-phase core–shell MENP system has an orders of magnitude higher ME coefficient compared to that of any single-phase multiferroic system.^10–12^ The core–shell MENP consists of a magnetostrictive core, cobalt ferrite, and a piezoelectric shell of barium titanate, which are lattice-matched at their surface interface. Upon application of a magnetic field, a strain propagates through the interface from the core to the shell, leading to generation of a dipole electric moment in the shell, thus inducing a local electric field in the nanoparticle's vicinity (Fig. 2a). This effect, known as the direct ME effect is the driving force of the MENPs’ therapeutic applications. For example, it can be used for local wirelessly controlled cellular modulation, *e.g.*, to induce local neural activity deep in the brain, for peripheral nerve stimulation,^13^ targeted drug delivery and release on demand, neurogenesis, trigger wireless irreversible electroporation (IRE) for high-specificity cancer therapy, or another therapeutic function.^14–17^ Conversely, upon application of a local electric field, a strain propagates through the interface from the shell to the core, thus leading to a magnetization change that can be detected remotely using a magnetic field sensor (magnetometer) (Fig. 2b).^18^ This effect, known as the converse ME effect, is the driving force of the MENPs’ diagnostic applications. For example, the converse effect could be used for wireless recording of any local electric field change due to cellular activity, whether it is due to neural firing, a difference in the dielectric properties between different cell types, or another function. In turn, the resulting nanoparticles’ magnetic moment change can be detected *via* functionalized magnetic resonance imaging (MRI), magnetic particle imaging (MPI) response, or another magnetometer-driven imaging.^19–25^

The materials properties of the core–shell MENPs of different compositions have been comprehensively characterized in many papers and reviews.^9,12,44^ However, in these studies, the nanoparticles’ key properties, *e.g.*, the ME coefficient, have been conducted mostly in a differential mode and did not take into account the highly non-linear physics characteristics of the two-phase nanoparticles, nor did they consider the cellular microenvironment effects on the magnetoelectric control of fundamental biological processes. This paper fills this gap.

### Nanoparticles need to be on membrane surface for direct interaction with the cell

**Fig. 3:**
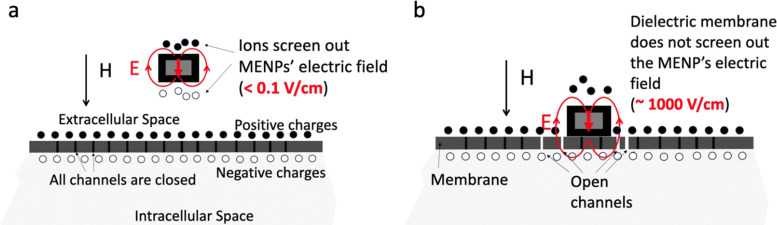
(a) Illustration of the field distribution around a MENP placed in the conductive extracellular space. Even in the saturated state, the electric field, *E*, is screened out by free ions with the sub-1-nm Debye length to a value below 0.1 V cm^−1^. The same is true if the nanoparticle is placed in the intracellular space. (b) In contrast, the same nanoparticle in direct contact with the dielectric membrane can generate a field on the order of 1000 V cm^−1^ across the membrane, with the electric field proportional to the applied magnetic field.

From the physics perspective, for both theranostic functions to be effective, MENPs need to be placed on the cellular membrane, except, arguably, for drug-delivery applications. A clear distinction must be made between two spatial biological domains determined by their conductive states: (1) conductive intracellular and extracellular spaces and (2) dielectric membranes. The membrane, made of many voltage-gated ion channels, separates the intracellular and extracellular spaces of each cell, thus representing an important hub that controls vital cellular signal pathways. At this relatively early stage of development, we are not discussing the very viable possibility of MENPs to interact with intracellular components such as mitochondria and others, with their own dielectric properties. For a nanoparticle to directly interface with this control hub, it needs to be on the membrane. Given the direct and converse ME coefficients on the order of 1 V cm^−1^ Oe^−1^ and 1 G cm V^−1^, respectively, significant local electric (∼1000 V cm^−1^) and magnetic fields (1 kOe) could be generated in the MENPs’ vicinity in response to application of magnetic and electric fields of reasonable, for theranostic purposes, strengths and frequencies. However, to fully benefit from this effect, it is crucial to ensure that the dielectric nanoparticle is in direct physical contact with the dielectric membrane. The key electric-field microenvironment difference between the nanoparticles not in direct contact and in direct contact with the membrane is illustrated in Fig. 3. If a MENP is located in either the conductive intracellular or extracellular space, and is not in direct contact with the dielectric membrane, the electric field generated by the nanoparticle in response to a magnetic field, even in a saturated (magnetization and polarization) state, would be screened out, by free ions, down to a relatively insignificant value on the order of 1 V cm^−1^, with a characteristic Debye length in the sub-1-nm size range (Fig. 3a). In contrast, if the same nanoparticle is located directly on the dielectric membrane surface, the field generated by the nanoparticle across the membrane could be as large as 1000 V cm^−1^ (Fig. 3b).^21^ In turn, such a strong electric field can locally activate ion channels in the membrane or even locally depolarize the membrane.

### Magnetic fields applied to control local electric fields need to account for magnetic core's non-linear *M*–*H* loop

Owing to the direct ME effect, the induced electric field, *E*, is proportional to the applied magnetic field, *H*. Hence, the impact of the effect can be controlled by the strength and frequency of the applied magnetic field. As a result, this system allows us to either modulate neural activity without damaging the cell, or irreversibly electroporate and kill a specific cells, depending on the strength of the magnetic field applied. It is important to note that it is the nanoparticles’ properties that determine critical limits such as a lower threshold below which is there is no effect as well as an upper limit beyond which no additional energy can be generated.

Arguably, the rectangular prism shape of MENPs, as illustrated in Fig. 3, might be preferred for several reasons, first and foremost including but not limited to maximizing the “useful” surface area for the required lattice-matched core–shell interface, as well as, improving the surface contact between the nanoparticles and the membrane.^29,33,34^

Again, the ME effect is the main property that underlies all the MENPs’ applications. Understanding how this effect works and how to properly control the effect for inducing a required treatment and/or diagnostic function is vital in developing these applications. This effect is often approximated by the linear phenomenological equation, derived from the expansion for the free energy as a power series of electric and magnetic fields:^45^

1 Δ*P*_*i*_ = *α*_*i*_*H*_*i*_,

where, *P*_*i*_ and *H*_*i*_ stand for the *i*-th components of the polarization and the applied magnetic field, respectively, *α*_*i*_ is the *i*-th diagonal term of the ME coefficient tensor, assuming zero cross-field terms. In turn, the induced local dipolar electric fields enable wirelessly controlled electric field modulation of cellular activity.^28,30,46^ The reciprocal version of this equation is used to describe the reading process with MENPs. Specifically, according to the converse ME effect, when exposed to a local electric field, the magnetization of these nanoparticles changes accordingly. In a similar linear approximation, the dependence is also extrapolated from the above LGD theory of multiferroics:^47^

2 Δ*M*_*i*_ = *α*_*i*_*E*_*i*_,

where *M*_*i*_ is the *i*-th component of the magnetization, is the *i*-th component of the applied electric field. Therefore, if MENPs are locally or globally distributed throughout the brain, the electric field due to neural firing in their vicinity will induce a non-zero magnetization change, which could be detected using sensitive magnetometers.

As mentioned above, the origin of the ME effect in the core–shell MENPs is due to strain propagation through the lattice-matched surface interface between the magnetostrictive core and the piezoelectric shell. Therefore, the temporal response of the core–shell nanoparticles is limited by the intrinsic physical resonances of both the core and shell, as well as the core–shell interface – the ferromagnetic resonance, the dielectric resonance and the mechanical resonance. Coincidentally, all these resonances take place in gigahertz ranges, *i.e.*, in the sub-ns time range, thus significantly exceeding the temporal response required for neural activity imaging in real time, *i.e.*, in the sub-ms range.^48–50^ However, because ideally the ME coefficient is the product of the magnetostrictive coefficient of the core and the piezoelectric coefficient of the shell, it is important to properly leverage the non-linear characteristics of these components, for example, the *M*–*H* hysteresis loop of the core.

**Fig. 4:**
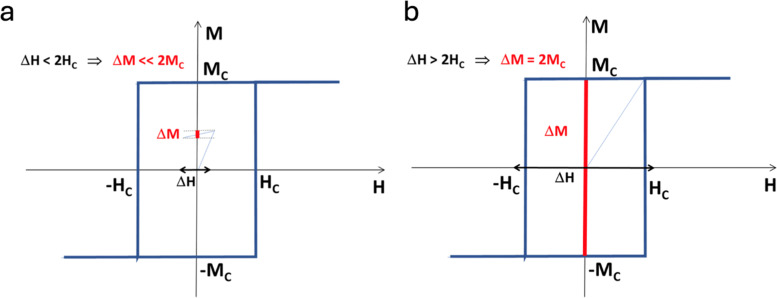
The magnetization, *M*, of the MENPs’ core depending on the applied magnetic field, *H*, for a simplified case of the *M*–*H* loop for the field orientation along an “easy” axis. No shape anisotropy is considered, assuming a relatively short measurement time. Two specific cases can be identified with respect to the ratio of the applied magnetic field change, Δ*H*, (shown by a thick black double arrowed line) to the coercivity field, *H*_C_ (<*H*_K_). (a) If the applied magnetic field is below the coercivity field, *H*_C_, the change of the magnetization, Δ*M* (shown by a red line), is relatively small compared to the saturation magnetization, *M*_C_, which in turn would result in a relatively small induced electric field. (b) If the applied magnetic field exceeds the coercivity field, the change of the magnetization becomes significant and reaches the doubled saturation magnetization: Δ*M* = 2*M*_C_.

It can be noted that the above LGD equation is an oversimplified approximation that assumes a strictly linear effect and does not account for intrinsic or extrinsic fields of the magnetic core in the core–shell MENPs.^28^ In turn, these fields are described by a highly non-linear and hysteretic *M*–*H* dependence known as the full *M*–*H* hysteresis loop. Below, it is shown why not considering the full *M*–*H* loop would lead to significant errors in predicting desired effects. For example, in the case of MENPs-based cellular modulation, the chronological sequence of the physical events to induce the ME effects in these core–shell nanostructures includes several distinct processes: (1) application of a magnetic field leads to a change of the magnetization, according to the *M*–*H* loop of the magnetic core; (2) the resulting change of the magnetization leads to a lattice parameter shift in the magnetic core because of its magnetostrictive effect; (3) because of the lattice-matched surface interface between the core and the shell, the same lattice parameter shift is transferred to the piezoelectric shell, causing strain propagation through the interface; (4) finally, because of the piezoelectric effect, the shell's lattice parameter shift induces a local dipole electric field. Hence, according to this scenario, to induce the largest possible electric field change, it is necessary for the magnetization of the core to experience the maximum possible change. The range of the magnetization change is defined by the hysteresis in the aforementioned *M*–*H* loop, as illustrated in Fig. 4. For the sake of simplicity, the illustration shows the *M*–*H* loop along an “easy” magnetic axis assuming only the contribution of the magneto-crystalline anisotropy, *i.e.*, without considering the shape anisotropy. The magneto-crystalline anisotropy field, *H*_K_, an intrinsic field determined by electron spin–orbit coupling, is the field that needs to be applied along a “hard” magnetic axis to fully align the magnetization along the field. The “hard” axis is defined as an axis perpendicular to the “easy” axis. The anisotropy field does not depend on extrinsic parameters such as crystalline impurities, temperature, and others. In contrast, the coercivity field, *H*_C_ (<*H*_K_) is an extrinsic field. It is defined as the field that needs to be applied against the magnetization direction to bring the magnetization value to zero. The coercivity field, always a fraction of the anisotropy field, depends on the relative orientation of the applied field with respect to the “easy” axis and other external factors, such as the quality of the material crystallinity, temperature, the measurement time, and others. According to an ideal single-domain uni-axial anisotropy approximation, the *M*–*H* hysteresis loop for different relative orientations of the applied field with respect to the easy axis is often described by the Stoner–Wohlfarth model.^51^ As described below in more detail, this field exponentially depends on the measurement time. If the measurement time for the field along the “easy” axis, is infinitely fast, the coercivity field is equal to the anisotropy field. In contrast, if this measurement time is infinitely slow, the coercivity field is zero.

This time dependence of the *M*–*H* loop is determined by the stability ratio, SR, *KV*/*k*_B_*T*, of the magnetic core.3
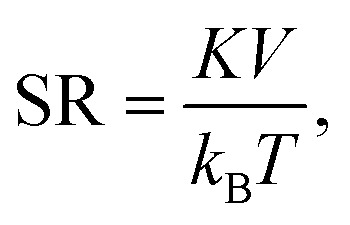
where, *K* and *V* are the core's magnetic anisotropy and volume, respectively, *k*_*B*_ is the Boltzmann constant and *T* is the ambient temperature. More specifically, the stability ratio determines the exponential time dependence of the non-volatility, also known as the shelf life, of the magnetic core,

4 *τ* ∝ *τ*_0_e^SR^,

where *t*_0_ is the characteristic time constant determined by the ferromagnetic resonance of the core material, typically on the order of 1 ns.^52^ According to this equation, the non-volatility time is exponentially dependent on the core size.^53^ To show how strongly this time depends on the nanoparticle's size, arguably the current most popular core–shell configuration of MENPs, *i.e.*, CoFe_2_O_4_@BaTiO_3_, consisting of the magnetostrictive core made of the inverse spinel cobalt ferrite crystal and the piezoelectric shell made of the perovskite barium titanite, is used as an example.^10^ Given the anisotropy of the cobalt ferrite on the order of 10^6^ J m^−3^, for simplicity assuming a cubic shape, reducing the core size from 10 nm to 7 nm would reduce the non-volatility time from over ten years to approximately 1 s. Further reducing the size to 6 nm would reduce the time into the sub-1-ms range. It can be noted that if an alternating current (AC) magnetic field is applied, the characteristic measurement time would be on the order of one half of the sine period, *T*/2 = 1/2*f*, where *T* and *f* are the AC field's period and frequency, respectively. Given the typical measurement frequency of 50 Hz, the characteristic measurement time is approximately 10 ms, therefore the 7- and 6-nm nanoparticles would be in the hysteresis and superparamagnetic states, respectively. It can be reminded that the magnetoelectricity in the aforementioned core–shell nanostructures cannot be maintained in the superparamagnetic state, because, given a perfect lattice-matched coupling between the core and the shell, the magnetoelectric coefficient is just a product of the magnetostrictive coefficient of the magnetic core and the piezoelectric coefficient of the ferroelectric shell. Therefore, because the magnetostrictive effect is not feasible if the electron spin and orbit are not connected, the magnetoelectric effect also cannot exist in the superparamagnetic state.^10^ However, the applied field frequency (the measurement time) and the size of the nanoparticles can be adjusted so that the nanoparticles do not fall in the superparamagnetic state, thus maintaining their significant magnetoelectric effect. In turn, this dependence of the superparamagnetic–ferromagnetic state transition on the size and the frequency of the applied field could be exploited as an “On/Off” switch of any function pursued with MENPs, whether it is cell stimulation, neural recording, drug delivery, or another.

**Fig. 5:**
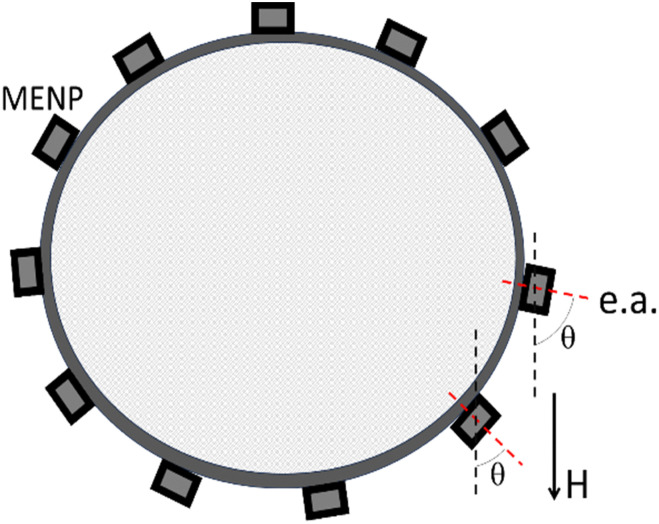
Spherical cell with MENPs uniformly distributed over the membrane surface. The angle (*θ*) between the applied field and the easy axis (e.a.) of a MENP, defined by the magnetic anisotropy of its core, varies from 0 to 360 degrees over the surface.

Another important observation from the full *M*–*H* hysteresis loop relates to the requirement of the applied field strength. To fully exploit the magnetoelectric effect, the applied field should be compared with the intrinsic anisotropy field, not with the extrinsic coercivity field. Assuming nanoparticles are evenly distributed on the cell's membrane surface, there is a 360-degree distribution of the angle between the applied field and the magnetic “easy” axis (e.a.) orientation, defined by the magnetic anisotropy, often dominated by the magneto-crystalline anisotropy contribution, as shown in Fig. 5. The anisotropy field, *H*_K_, does not depend on this angle; in contrast, the coercivity field, *H*_C_, varies from 0 to *H*_K_, depending on this angle. Hence, assuming MENPs are attached to the membrane, to ensure all the nanoparticles’ magnetic moments are aligned along a uniform applied magnetic field, the applied field should exceed the anisotropy field. Otherwise, only a fraction of the nanoparticles, specifically only those with their axes *a priori* oriented along the applied field, will go through the full *M*–*H* hysteresis loop of the magnetostrictive core. In turn, only this small fraction of nanoparticles will display the maximum possible electric field variation.

Using neural stimulation as an example, the above physics can be applied to derive a phenomenological expression to describe how the MENPs’ physical properties, *e.g.*, the core’ anisotropy and size, could be used to wirelessly control neural activation. Again, it is assumed that the nanoparticles are in direct contact with the membrane, and therefore become an integral part of the membrane. Also, it is assumed that there are a sufficient nanoparticles that together deliver enough energy to the neuron, to induce an action potential. In this case, the probability of inducing a neural firing event, *P*, would be equivalent to the probability of a full reversal of the nanoparticle's magnetization, *P*_M_. To simplify the analysis, a cylindrical anisotropy of the magnetic core is assumed.^54^5
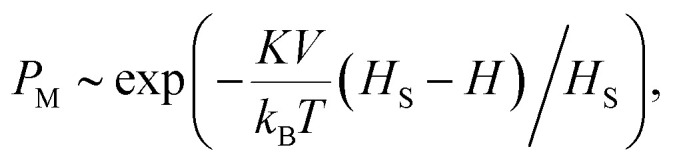
where *H*_S_ is the characteristic field that is required to saturate the average nanoparticle in the system, *H* is the applied field.

Assume the surface density of the nanoparticles required to provide enough energy to overcome the firing threshold is *n*_Thr_. This threshold depends on local microenvironment conditions, *e.g.*, pH level, temperature, neural type and density, and others. Then, the probability for a neuron to fire could be evaluated as6
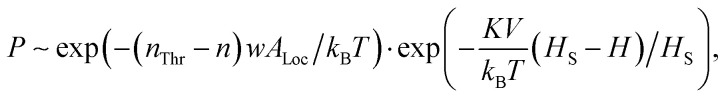
where *n* is the surface density of nanoparticles, *A*_Loc_ is the surface of the selected local region on the membrane, *w* is the energy transmitted to the neuron by the nanoparticle due to the ME effect. The expression is valid for *n* < *n*_Thr_ and *H* < *H*_S_.

Again, it is important to note that the above analysis is valid assuming a single-domain approximation. However, this single-domain approximation holds only for sufficiently small size nanoparticles. In turn, the relative threshold size depends on the interplay between the domain wall energy and the demagnetization energy, thus being specific to intrinsic material properties such as the magneto-crystalline anisotropy energy density, *K*, the spin-exchange constant, *A*, and the saturation magnetization, *M*_S_. According to a trivial physics analysis, approximating the nanoparticle as a sphere, the single-domain threshold size, *δ*_th_, can be evaluated to be on the order of:7
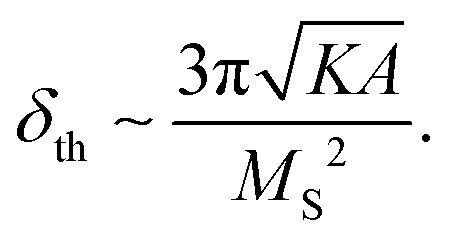
For example, assuming typical values for cobalt ferrite, *i.e.*, *K* ∼ 10^6^ erg per cc, *A* ∼ 10^−6^ erg per cm, and *M*_S_ ∼ 60 emu per cc, this threshold thickness would be on the order of tens of microns, which is significantly beyond the size of a nanoparticle of interest, *i.e.*, in the sub-50-nm size range, the range at least partially determined by the generally perceived size limitation to cross the BBB.^55^ Therefore, under a global equilibrium condition, the nanoparticles in this size range could be considered in a single domain state. However, given the relatively high anisotropy of this material, a local equilibrium can be maintained assuming the local size is larger than the domain wall thickness. The domain wall thickness, *δ*_w_, could be evaluated according to this trivial one-dimensional approximation:8
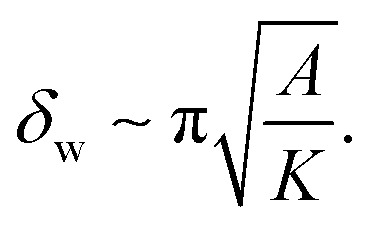
In zeroth approximation, neglecting a surface anisotropy at the nanoscale, for the above typical values of the cobalt ferrite's magneto-crystalline anisotropy energy density and exchange constant, this expression gives the wall thickness on the order of 30 nm. Hence, a cobalt–ferrite nanoparticle in the sub-30-nm size range is likely to be in a single-domain state even under local equilibrium conditions. This trivial analysis might be sufficient to estimate basic properties of the nanoparticles. However, for a more comprehensive analysis and a more reliable prediction, *e.g.*, to understand the spin geometry in the magnetostrictive core at the surface interface with the piezoelectric shell, it is important to calculate a geometry-dependent non-uniform distribution of adjacent spins. The latter is the purpose of nanomagnetic simulation, considering quantum–mechanical interactions between adjacent spins.^56^ The nanomagnetic simulation would be particularly important to further significantly improve or optimize the MENPs’ performance by tailoring material properties to each specific application, whether it is neural modulation, irreversible electroporation, targeted delivery, neural recording, or a combination of any of the above. Even according to the above trivial analysis, it can be noted that any function of these nanoparticles can be finely tuned to match a specific application through variations of their intrinsic properties, such as, the magnetocrystalline anisotropy energy, the exchange constant and the saturation magnetization, as well as their sizes and shapes. For example, controlling the composition of the core material alone is a powerful control knob. Considering the above core–shell MENPs, the core is made of a ferrite, for example, cobalt ferrite, ideally in an inverse spinel lattice configuration. Ferrites have a spinel configuration, with oxygen atoms forming a close packed face-centered cubic (FCC) structure, with two types of cation ions, divalent and trivalent, occupying interstitial sites of this FCC structure. There are two types of interstitial sites, tetrahedral (“A”) and octahedral (“B”). The inverse spinel is formed when all the divalent ions are placed on the octahedral sites, while the trivalent ions equally divided between the tetrahedral and octahedral sites. The magnetic states of “A” and “B” sites depend on the cations used to form the inverse spinel. In turn, this allows for a significant diversity of properties that could be tailored through a compositional change. For example, besides the three magnetic elements of Fe, Co and Ni, other transition metals, metalloids, and post-transition metals which could be used to form cations with unpaired spins in the d sub-shell include Mn, Cu, Mg, Ge, Al, Gd, Li, Sn, Ti, Zn, Cd, and others.^57^ The magnetic anisotropy energy in these materials is due to the spin–orbit coupling, thus following the crystallographic FCC symmetry. The dominant coupling between any two adjacent A and B spins is due to the super-exchange interaction *via* a 3p orbit of the intermediate oxygen, thus always leading to an anti-parallel spin coupling, in turn resulting in a ferrimagnetic order, given the A and B spins have different amplitudes. It is important to ensure that the synthesis process can produce the desired inverse spinel compound in a ferrimagnetic state, opposed to any one of the many other possible and structurally similar compounds, such as the antiferromagnetic hematite and others. In other words, all the three intrinsic properties of these materials, *i.e.*, the anisotropy energy, the exchange constant and the saturation magnetization, respectively, can be controlled in a wide range just through selection of the cations in the inverse spinel structure. However, it should be reminded that this analysis is oversimplified for the sake of explaining the importance of the choice of material composition, nanoparticle size, and shape. For example, as mentioned above, it does not consider the surface anisotropy effects that can be relatively significant in these material systems at the nanoscale.^58–60^ These surface effects, which strongly depend on the composition, size and shape, might play a dominant factor in determining the observed effects and even the main magnetoelectric effect. For example, there have been experimental and theoretical studies to demonstrate that the surface of ferrites could transition into a conducting ferromagnetic state if the characteristic size is reduced into the sub-10-nm range.^61,62^

As mentioned above, the ME effect in these core–shell nanostructures is due to a lattice match between the magnetostrictive core and the piezoelectric shell. Hence, the ME effect is maximized when the following three conditions are optimized: (i) the magnetostrictive effect of the core reaches its maximum in the field range under study, (ii) the piezoelectric effect of the shell reaches its maximum in the field range under study, and (iii) there is a perfect lattice match between the core and the shell along certain crystallographic orientations at the surface interface between the two components. We discussed the first condition above. The second condition is analyzed below. As for the third condition, from the physics argument of energy minimization, the best lattice match between the inverse spinel structure of the cobalt ferrite core and the piezoelectric tetragonal polymorph of the barium titanite shell would likely happen at an interface with the ratio between the respective lattice parameters of 2 to 1.^63^ In turn, such an interface would lead to creation of core–shell nanoparticles with a rectangular prism shape.^29,33,44^

Generally, for some of the highest performing piezoelectric materials, both high mechanical coupling coefficients *k* and piezoelectric coefficients d_*xy*_, are in the family of lead-zirconate titanate (PZT) and other lead derivatives. While ideal in terms of piezoelectric characteristics, these materials are not practical for use in a BMI or any other medical application, as lead is a well-known toxin to both the human body and environment. Biocompatible alternatives are widely available, at the cost of comparative performance to their lead counterparts. The added benefit of these biocompatible piezo materials is that they offer an increased range in magnetostrictive core choice. The aforementioned cobalt ferrite, for instance, is a known health hazard,^64^ making magnetostrictive metal oxides such as cobalt ferrite normally impossible for use in medical applications. However, complete coverage with a piezoelectric shell material that is biocompatible would increase the number of viable options for such core materials. Barium titanate (BaTiO_3_) and its derivatives, such as BZT (BaZr_*x*_Ti_(1−*x*)_O_3_), various niobates such as potassium sodium niobate (KNN) and lithium niobate (LN), and other ceramic oxide/nitrides are biocompatible piezoelectric coatings.^65–68^ Barium titanate is an extensively studied material, along with BCZT (Ba_0.85_Ca_0.15_Zr_0.1_Ti_0.9_O_3_) and other doped variants. However, considering this material system, an important condition to bear in mind is to ensure the component's tetragonal *versus* cubic phase for maximizing the piezoelectric effect.^69,70^ The requirement to minimize the unfavorable transition (from tetragonal to cubic phase) places certain temperature constraints during the crystallographic growth, *e.g.*, cooling through 120 °C.^71,72^ Furthermore, it is feasible that the appropriate magnetostrictive core's shape, *e.g.*, of a rectangular prism, would promote the growth of the tetragonal phase of the piezoelectric shell.^29^ Polymer piezoelectrics do exist, with polyvinylidene fluoride (PVDF) as one such example.^73^ The piezoelectric performance is generally lower than that of their ceramic counterparts, but it still can be adequate, nonetheless. The risk with polymer coatings, more so than with harder materials such as ceramics, is that of degradation/decomposition in the body rendering the surface of possibly toxic core materials exposed. This could be avoided by using biocompatible or biodegradable magnetostrictive materials, however, with the loss of the piezoelectric shell, the key magnetoelectric effect of these nanoparticles is also lost.

### Using magnetic field frequency to control excitation and inhibition of neural activity

**Fig. 6:**
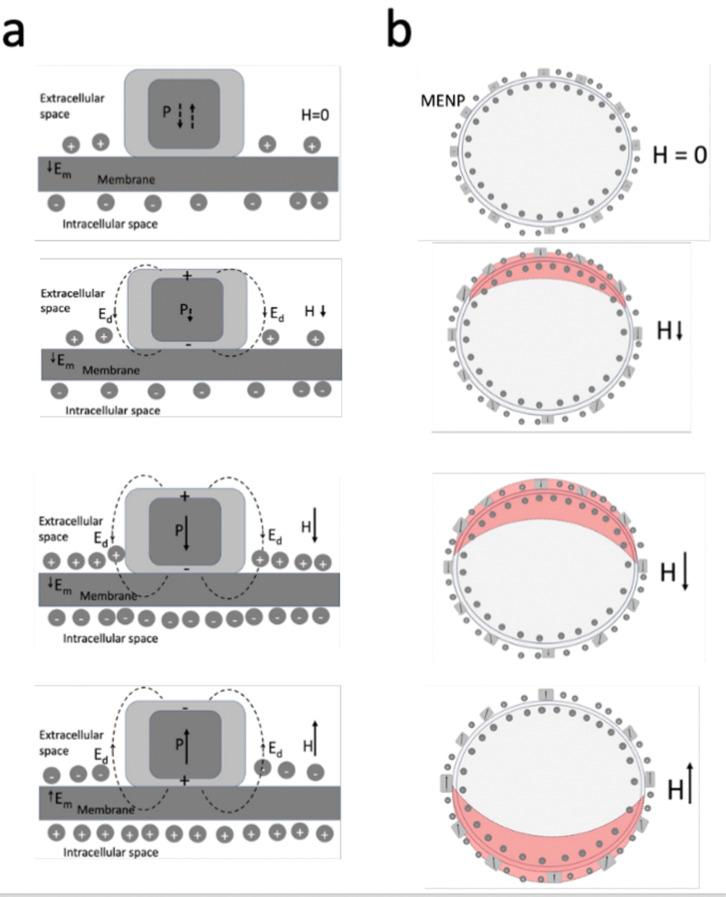
Application of AC and DC magnetic fields could break the typical spherical symmetry of the neural firing process. (a) High-level illustration showing how application of an AC magnetic field, with a frequency, *f*, chosen so that the effective measurement time, 1/*f*, is comparable to the characteristic ion channel activation time, application of the AC magnetic field effectively creates a resonance condition, in turn causing a local neural excitation: (top) no field applied, the nanoparticle remains in a relatively depolarized state, only with a slight non-zero polarization, the membrane potential is close to its resting value; (middle) negative magnetic field orientation polarizes the nanoparticle's electric dipole, the nanoparticle's electric field further increases the membrane potential; (bottom) reversing the magnetic field orientation polarizes the nanoparticle's electric dipole moment in the opposite orientation to induce an electric field that locally depolarizes the membrane, thus effectively reducing the local membrane potential. (b) Illustration to show how application of a DC magnetic field can inhibit neural activation by breaking the typical spherical symmetry of the neural firing process. The top image shows the average neuronal cell with MENPs lined up uniformly and symmetrically with respect to the spherical shape at zero field, *H* = 0. At zero field, the magnetic core of each MENP might be close to being demagnetized. As a non-zero field is applied, the symmetry is broken along the applied field direction. Depending on the field orientation, either the top or bottom part of the cell is experiencing a significantly increased effective membrane potential (shown by red regions), thus significantly reducing the ability to form an action potential.

Basic physics can be used to conceptually explain how application of AC and DC magnetic fields could lead to excitation and inhibition of neural activation, respectively, assuming the nanoparticles are uniformly placed on the neuronal membrane surface. Fig. 6a shows a high-level illustration of a typical modulation process by one nanoparticle. As mentioned above, when placed on the membrane, the MENP becomes an integral part of the membrane. Therefore, upon application of a magnetic field, due to the ME effect, the induced electric field in the immediate vicinity of nanoparticles changes accordingly, thus also effectively changing the local membrane potential. If the AC field frequency, *f*, is chosen so that the characteristic measurement time, 1/*f*, is comparable to the characteristic ion channel activation time, the application of the AC field would effectively create a resonance condition promoting neural activation^74,75^ In the other extreme, Fig. 6B shows how application of a DC magnetic field could cause an inhibition of local neural activity by breaking the typical spherical symmetry of the neural firing process.

According to the described basic physics model, the ability to control physical properties of MENPs can unlock a dynasty of novel biomedical applications. Below, a few examples are presented to show how controlling certain nanoparticles’ properties could enable specific applications. Certain requirements on MENPs are common for all these applications. For example, as described above, for MENPs to enable wireless control of fundamental biological processes, it is vital to place these nanoparticles at the cellular membrane. By placing MENPs on the membrane, they effectively become an integral part of this important control gate and are able to control this gate wirelessly and locally. Every related application would significantly benefit from this requirement being met. However, there are certain requirements on the MENPs’ properties that would be specific to each application, as discussed below through a few examples.

## Theranostic applications: challenges and potential solutions

### Biosafety, delivery, biodistribution and clearance

The described core–shell MENPs have been extensively studied for biosafety and toxicity limits and are considered safe even at dosage levels significantly higher than that typically used in many recent studies, *i.e.*, on the order of 1 μg per the average rat brain.^30,46,76–80^ Several experiments have been conducted to show delivery of these nanoparticles deep into the brain across the blood–brain barrier (BBB) *via* several administration routes including intravenous (IV) injection and intranasal (IN) inhalation as well as direct injection into target sites.^30,46,77,81,82^ To understand the nanoparticles’ size-dependent biodistribution and clearance, a comprehensive study based on an elemental compositional analysis with the energy-dispersive spectroscopy (EDS) mode of scanning electron microscope (SEM) imaging of many tissues from different organs was conducted in mice; the study showed that that most nanoparticles are excreted within a 2-month period, unless a reversed field gradient is applied to pull the nanoparticles back to the blood circulation, thus expediting the clearance process.^83^ Recent studies from independent laboratories have also shown that MENPs are nontoxic to brain and other major organs and do not affect hepatic, kidney, and neurobehavioral functioning.^30,31,46,76,77,83^

### Therapeutics: neural modulation

**Fig. 7:**
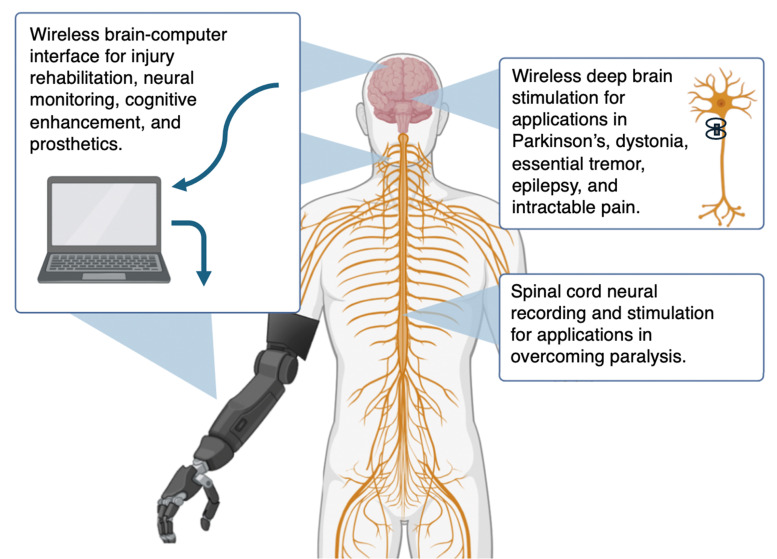
Diagram showing some examples of the applications of MENPs in neural modulation. In one embodiment, the required magnetic field sources and detectors to control MENPs would be embedded into a special light-weight helmet.

In this application, MENPs are expected to locally modulate neural activity *via* application of magnetic fields, without causing any irreversible damage to the cellular microenvironment (Fig. 7). The idea of using MENPs for the purpose of wireless non-invasive deep-brain stimulation was for the first time proposed in a theoretical paper by Yue *et al.* in 2012.^1^ In 2021, the first experimental demonstration where they used MENPs to stimulate neural activity in the mouse brain was described in a paper by Kozielski *et al.*^30^ Also in 2021, the first experiment where they used two-photon (2P) imaging to correlate imaged neural activity in the mouse brain, with intravenously administrated MENPs, with application of a sinusoidal magnetic field was described in a paper by Nguyen *et al.*^46^ In 2021, the first application to use the MENPs’ ability to wirelessly stimulate cells for the purpose of neural regeneration through an *in vitro* experiment was described in a paper by Zhang *et al.*^78^ In 2022, the first *in vitro* experiment to show neural firing in E18 rat hippocampal cell cultures, synchronized with application of a magnetic field with a sub-20-ms precision was described in a paper by Zhang *et al.* In 2022, a theoretical study on modeling MENPs for biomedical applications, with a focus on modulation, was described in a paper by Fiocchi *et al.*^84^ In 2023, a study that demonstrated MENPs-based modulation *in vitro* and *in vivo* was also described in a paper by Kim *et al.*^29^ In this study, they for the first time used rectangular shape MENPs to promote the ME coupling to the membrane. Furthermore, in the two mentioned experimental studies by Zhang *et al.* and Kim and *et al.*, respectively,^28,29^ the concentration of the nanoparticles used, on the order of 1 μg of MENPs per 100 000 neurons, was approximately two orders of magnitude less that that used in previous experiments,^30,36^ arguably, because of significantly improved attachment of the particles to the membrane surface, whether it is due to surface functionalization or optimizing the shape of the nanoparticles. In 2023, a theoretical study to numerically simulate the MENPs-based modulation to show the importance of the nanoparticles’ shape was described in a paper by Marrella *et al.*^33^ In 2023, an *in vivo* study to show how MENPs could be used for targeted wireless stimulation of the subthalamic nucleus to modulate key monoaminergic systems, like in contemporary deep brain stimulation applications, was described in a paper by Alosaimi *et al.* In 2022, another important *in vivo* study on mice to demonstrate the ME-induced local electric field effects of MENPs to dissociate highly stable A beta aggregates in the Alzheimer's disease (AD) mouse brains under application of AC magnetic fields was described in a paper by Jang *et al.*^31^ In 2024, an innovative implementation in which MENPs were uniformly embedded into a sheet of foldable paper with the purpose of creating a magnetically controlled bioelectronic implant for neuromodulation of different shape tissues was described in a paper by Choe *et al.*^85^ In 2024, multiscale modeling of MENPs for the analysis of spatially selective neural stimulation was described in a paper by Kumari *et al.*^86^ In 2024, for the first time, a field-synchronized generation of action potentials, not just sub-threshold activation, was demonstrated in an *in vitro* study by Zhang *et al.*^44^ This last study also for the first time experimentally showed how application of a D.C. magnetic field could be used to inhibit local action potentials. In a recent study by Ye *et al.* (2024), they conducted both *in vitro* and *in vivo* studies on a zebra fish spinal cord injury (SCI) model to demonstrate how integration of human-induced pluripotent stem cell-derived neural progenitor cells (NPCs) with MENPs could ensure rapid differentiation of NPCs and their integration into damaged neural pathways, significantly enhancing neural regeneration *via* application of AC magnetic fields.^87^ In 2024, a theoretical study on the effects of MENPs’ properties and relative field orientations on peripheral nerve stimulation was described in a paper by Galleta *et al.*^88^ In 2024, two theoretical studies to understand the MENPs’ contribution into neural circuits through drawing equivalent circuits was presented by Ramezani *et al.*^89,90^

Assuming MENPs are placed on the neuronal membrane, they need to generate a moderately high local electric field across the membrane to be able to modulate the local neural activity, while not causing any damage to the membrane. In this context, the moderately high electric field implies a field of ≪0.01 mV nm^−1^ (100 V cm^−1^). Arguably, the field on this order was shown to be utilized in the mentioned *in vitro* and *in vivo* experiments with MENPs.^28,30,36,46,91^ In all these experiments, the main focus was to wirelessly modulate neural activity through generation of a local electric field, while minimizing or entirely eliminating any potential mechanical effects due to a collateral application of a force to move the nanoparticles away from the membrane. Indeed, such a force could be present because of the presence of both magnetic field gradient and nanoparticles’ non-zero magnetic moment. It might be difficult to entirely eliminate the magnetic field gradient, with the magnetic field source located outside the brain. However, the nanoparticles’ remanent magnetic moment could be significantly minimized, while maintaining a relatively high ME effect, through a synthesis process, as described above in more detail.^28^ For example, the MENPs’ saturation magnetization was on the order of 1 emu g^−1^ in the study by Zhang *et al.*, compared to a typical value for the cobalt ferrite of 50 emu g^−1^.^28^ In this case, the magnetic force acting on a 30-nm nanoparticle due to a typical magnetic field gradient on the order of 1000 Oe cm^−1^ could be neglected compared to the van der Waals force holding the nanoparticle attached to the membrane.^53^

It can be noted that if the modulation is expected to be a reversible process, it might not be desirable to use the full electric-field-generation potential of MENPs. Hence, MENP compositions with a lower ME effect, compared to the most widely used CoFe_2_O_4_@BaTiO_3_, could also be used. For example, NiFe_2_O_4_@BaTiO_3_ might be more preferred in this case. Indeed, the magnetostriction coefficient of the nickel ferrite is an order of magnitude smaller than that of the cobalt ferrite, thus resulting in the ME coefficient being also an order of magnitude smaller. In this case, application of a 1000-Oe magnetic field would lead to an electric field on the order of 100 V cm^−1^ in the immediate proximity of the nanoparticle, which in turn, if the nanoparticle is in direct contact with the membrane, would lead to the same order of magnitude field applied across the membrane. Such a field is ideal to modulate neural activity, without causing any potential IRE-induced irreversible damage to the membrane.^92^ In summary, arguably, it would be rational to ensure that the electric field applied by the nanoparticle in contact with the membrane is strong enough to modulate local neural activity, while not too strong as to electroporate the membrane; this could be achieved either through controlling the applied magnetic field and/or tailoring the MENPs’ composition, size and shape.

### Therapeutics: cell electroporation

**Fig. 8:**
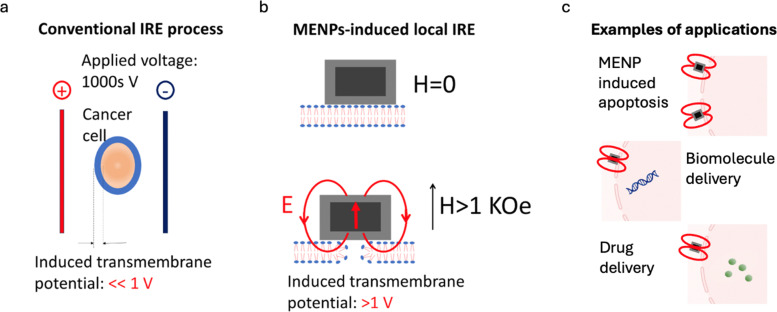
IRE process generated (a) in the traditional setting, and (b) in the MENPs-based highly localized, wirelessly induced, IRE. (c) Applications include inducing apoptosis in cancer cells (irreversible electroporation) as well as biomolecule and drug delivery (reversible electroporation). The illustrations are not to scale.

Electroporation is a process that employs relatively high electric fields, on the order of 1000 V cm^−1^, to reversibly or irreversibly porate cellular membranes with the purpose of controlling fundamental biological mechanisms at the cellular level.^92,93^ Depending on the applied electric field strength, the process can be reversible or irreversible. At smaller fields, ≪1000 V cm^−1^, the process is reversible and can be used for high-specificity targeted delivery of RNAs and other biomolecules designed to inhibit cancer cell proliferation.^92^ At stronger fields, >1000 V cm^−1^, the process becomes irreversible and can be used to rupture cell membranes *via* a dielectric breakdown of the lipid bilayer, consequently unleashing a relatively strong immunological response.^94^ This cut-off between the reversible and irreversible processes is determined by the size/number of pores generated in the membrane when a threshold is reached beyond which they are not able to be repaired. Furthermore, because different cell lines have membranes with different dielectric properties, electroporation opens a pathway to high-specificity treatment. For example, the process of IRE shows great promise to treat cancers that cannot be treated by other ablation techniques.^95^ Indeed, IRE has been shown to enhance the body's ability to kill cancer cells that survived the initial treatment. However, despite this therapeutic ability, traditional IRE, due to its need for physical electrodes and generation of relatively high electric fields, remains an invasive procedure for only medically fit patients and is associated with considerable toxicities to sensitive nearby normal tissue.^14,96^ In contrast, MENPs, due to their ability to induce relatively strong and highly localized electric fields across the membrane, hold promise to achieve similar therapeutic effects while eliminating the risks inherent in traditional IRE. Furthermore, it is important to note that there is a significant quantitative advantage of the MENPs-based IRE process over the traditional IRE process. For example, in the traditional process (Fig. 8 left), to induce electroporation in an extremely local region, *i.e.* across the ±10 nm membrane of the cancer cell, they apply 1000 s of volts to the region between positive and negative electrodes separated by at least a few millimeters. Because the cellular media is mostly conductive, the resulting induced electric field across the membrane would be on the order of 1 V cm^−1^, despite the fact the voltage applied is on the order of 1000 s of volts. In contrast, with the MENPs-based IRE process (Fig. 8 right), when exposed to a relatively weak magnetic field, on the order of 1 kOe, the nanoparticle induced electric field across the membrane would be on the order of 1000 V cm^−1^, orders of magnitude higher than that in the traditional IRE process. As a result, with the MENPs-based IRE process, there is no need to apply a magnetic field in the AC mode and with a relatively short pulse width (<0.1 ms) to deliver the energy required for inducing IRE; in other words, even a DC magnetic field could cause the required dielectric breakdown across the cancer cell membrane. Again, here it is assumed that the nanoparticle is placed directly on the membrane surface. However, it is known that the IRE process depends on the applied field frequency. Indeed, the approach known as the high-frequency IRE (H-FIRE) suggests that the field strength threshold reduces at higher frequencies.^97^ Hence, while a DC field may be sufficient now, the approach could be further improved at higher frequencies.

The concept of the MENPs-based nano-electroporation was for the first time proposed in a paper by Guduru *et al.* in 2013.^15^ Other *in vitro* studies to study nano-electroporation in response to application of AC magnetic fields were described in a paper by Betal *et al.* in 2016 and a paper by Kaushik *et al.* in 2017.^16,98^ The first *in vivo* study where they used nano-electroporation for targeted drug deliver to treat ovarian cancer in mice was described in a paper by Rodzinski *et al.* in 2016.^43^ An vitro study to understand the effects of MENPs on colon cancers, where they comparatively studied a range of doped core–shell nanocomposites such as Co_0.8_Mn_0.2_R_0.02_Fe_1.98_O_4_@BaTiO_3_ nanocomposites (R = Ce, Eu, Tb, Tm, or Gd), was described in a paper by Alfareed *et al.* in 2022.^99^ In addition, the same team studied and demonstrated biocompatibility of these nanoparticles in another paper in 2022.^79^

Compared to the above application to use MENPs for wireless modulation of neural activity, the current application to use MENPs for cell electroporation requires generation of an electric field at least an order of magnitude higher (on the order of 1000 V cm^−1^*versus* 100 V cm^−1^). Hence, MENPs with the highest possible ME effect must be used. To date, arguably, the above CoFe_2_O_4_@BaTiO_3_ nanoparticles display the highest measured ME coefficient, on the order of 1 V cm^−1^ Oe^−1^.^10^ Hence, when exposed to a magnetic field exceeding the anisotropy field of their cobalt ferrite core, *i.e.*, on the order of 10 kOe, these nanoparticles’ magnetization would be saturated along the field orientation, thus generating a local electric field across the membrane on the order of 1000 V cm^−1^.

### Therapeutics: targeted delivery

**Fig. 9:**
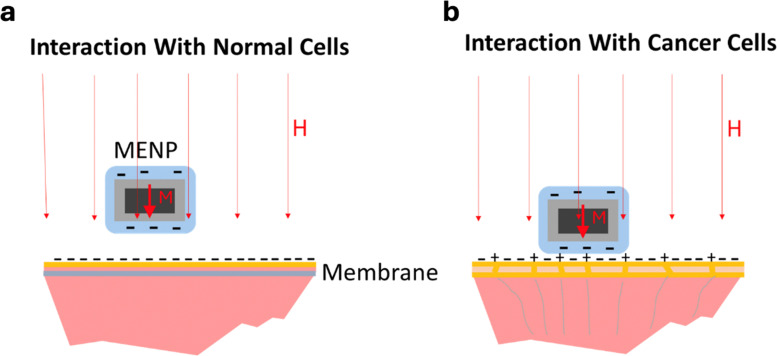
High-specificity targeting by MENPs. (a) Normal cells have polarized membranes, with a negatively charge surface in the extracellular space. Because a MENP is also negatively charged, the electrostatic Coulomb force is repulsive. (b) The repulsive electrostatic force is significantly reduced if a MENP is interfacing a strongly depolarized cancer cell membrane. As a result, the probability of overcoming the repulsive electrostatic force and reaching the region of the membrane proximity dominated by the attractive van der Waals force is significantly higher for the cancer cell. Application of a magnetic field localizes MENPs within the non-zero field region, because of their non-zero magnetic moments. This allows the MENP to be localized, in the cellular microenvironment, on the cancer cell membrane to induce, for example, irreversible electroporation leading to cellular apoptosis, or high specificity biomolecule and drug delivery to cancer cells, while excluding healthy tissue.

One of the most viable applications of MENPs is high-specificity targeted delivery.^100^ Because MENPs have a non-zero magnetic moment, they can be physically “pulled” to a target site, especially with an aid of an image-guided modality such as MRI or MPI. Furthermore, owing to their ME effect, MENPs offer other independent routes for targeted, high-specificity, delivery. One approach, in which the ME effect is used to control the overall surface charge of the nanoparticle, thus tailoring the electrostatic force that contributes to the interplay of the attraction and repulsion forces in the nanoparticle–cell interaction, was described in a paper by Stimphil *et al.* in 2017.^53^ In general, the two key, opposing, contributions to this interaction come from the net electrostatic force and the attraction van der Waals force, with the electrostatic force being either attractive or repulsive depending on mutual electrostatic states of the nanoparticle and the cell membrane. It has been established through zeta potential experiments that MENPs, coated glycerol monooleate (GMO), can have negative surface charge.^43^ Hence, driven by the random Brownian motion, when MENPs get close to the negative charged surface of the normal cell's polarized membrane, the electrostatic force is repulsive. However, if nanoparticles get close to the less charged surface of the cancer cell's depolarized membrane, the electrostatic repulsion is significantly reduced. As a result, the probability for the nanoparticle to reach the region in the membrane surface proximity where the attractive van der Waals force dominates significantly increases for the cancer cell (Fig. 9).

As mentioned above, it is important to note that the nanoparticle's proximity to the membrane is vital for enabling all the promising biomedical applications. Otherwise, the electric fields generated by the nanoparticle would die off at the Debye length, which is significantly smaller than one nanometer for the conductive intracellular or extracellular media, thus rendering the nanoparticle's ME effect significantly less useful. This difference in the electrostatic force between a nanoparticle and normal vs cancer cells would exist also in the presence of a magnetic field. The main purpose of the magnetic field would be to contain the nanoparticle in the selected region around the cell for as long as necessary to enable targeting of the cancer cells, while sparing the normal cells, depending on a specific application.

In 2013, the application of using MENPs for targeted drug delivery was for the first time presented in a paper by Nair *et al.*^3^ They argued that due to the direct ME effect, application of AC magnetic fields could be used to control molecular-level electric fields involved in the bond between the nanoparticles and the bio-load, thus enabling a wireless control of the drug retention and release on demand *via* application of AC magnetic fields of certain strengths and frequencies. They demonstrated this concept experimentally using an *in vitro* model of the blood–brain barrier to use drug-coated MENPs to deliver retroviral therapy across the barrier and then successfully eradicate HIV-1 virus. The study was followed by other *in vitro* studies to show delivery of various bio-loads, *e.g.*, drugs, RNAs, peptides, and others, on different cell culture models.^15,76,101,102^ In 2014, different conjugation methods were explored to investigate the chemistry-dependent strength of the nanoparticle-bio-load bond and the bond's field sensitivity in a paper by Guduru *et al.*^103^ In 2016, the first *in vivo* study on mice to show how the same approach could be used to eradicate ovarian cancer in xenograft mice was described in a paper by Rodzinski *et al.*^43^ In 2019, the first *in vivo* study on non-human primates (NHPs) to use the MENPs’ approach for drug delivery across the blood–brain barrier was described in a paper by Kaushik *et al.*^77^ In 2021, an application of MENPs to deliver drugs for treatment of neurological tuberculosis and HIV was described in a paper by Mhambi *et al.*^104^ In 2024, a study on mice to show how MENPs could deliver drugs across BBB to enhance chemotherapy and reduce postoperative glioma recurrence was described in a paper by Huang *et al.*^105^ In all these experiments, the applied AC field had a relatively low strength (≪1 kOe) and a relatively low frequency (<1 kHz).

In this application, it is important to ensure that a bio-load is attached to MENPs as tightly as possible to ensure optimal drug retention until the nanoparticles reach the target site(s) and the bio-load can be released on demand *via* application of a magnetic field with a specific spatiotemporal pattern, thus avoiding off-target delivery and potential side effects. This field-controlled retention switch provided by MENPs due to their ME effect is a very promising property for future on-demand drug release applications. However, more *in vitro* and *in vivo* studies will need to be conducted to compare different drug-nanoparticle conjugation approaches with respect to the field-application control. Furthermore, extending this technology to a broader frequency range (≫1 kHz) might further significantly improve the targeted delivery approach.

### Diagnostics: reading back cellular activity *via* magnetic field imaging techniques such as magnetic resonance imaging and magnetic particle imaging

**Fig. 10:**
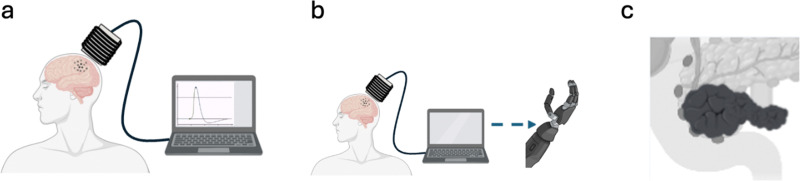
Some application examples using MENPs ability to read back information from the cellular environment. (a) Tracking brain electrical activity in diseases such as Parkinson's and epilepsy, and (b) relaying neuronal information to prosthetic limbs or other brain computer interface devices, using the converse of the ME effect. In addition, (c) using MENPs as an MRI contrast agent due to the magnetic properties of the cobalt ferrite core, highlighting tumor tissue in the body. The MENPs-enhanced MRI signal contains additional information because of the nanoparticles’ sensitivity to local electric fields, *e.g.*, at the cellular membranes, thus providing additional specificity to cell types, compared to the conventional magnetic nanoparticles enhanced MRI signal.

The MENPs-based cellular recording – the mode reciprocal to the above modulation mode – is a more challenging task because the requirements on the nanoparticles’ non-linear properties are significantly more stringent compared to those for the modulation mode, as discussed below in more detail (Fig. 10). To date, the mode of recording with MENPs is at its most nascent and primarily theoretical stage, with no experiments conducted yet to prove the concept with adequate statistical significance. The idea of using the converse ME effect of MENPs to read back electric-field-based information from a cellular microenvironment was for the first time proposed in a paper by Nagesetti *et al.* in 2017.^106^ Particularly, they used MENPs to modify the nuclear magnetic resonance (NMR) spectra which were wirelessly read back from different cell lines using a low-field (∼2 kOe) NMR detector. They hypothesized that by placing MENPs directly on the membrane, the nanoparticles were exposed to the relatively strong electric fields across the membrane, thus changing the magnetic moment of the nanoparticles because of the converse ME effect. In turn, the changed magnetic moment could be detected through modulation of the measured cell line NMR spectrum. Furthermore, they argued that because different cells, *e.g.*, healthy and cancer ovarian and breast cancer cells, normal endothelial and glioblastoma cells, had different electric fields across the membrane, MENPs should differently modulate the measured NMR spectra. In other words, they hypothesized that the modulated NMR spectra of cells with MENPs were specific to the measured cell lines. They also conducted scanning probe microscopy (SPM) measurements, including atomic force microscopy (AFM) and magnetic force microscopy (MFM), to confirm that the nanoparticles were attached to the membrane. A COMSOL-based simulation to show how MENPs could be used to map electric fields in the brain when integrated with MRI was discussed in a paper by Guduru *et al.* in 2018.^19^ The first *in silico* study in which they simulated neural recording with MENPs integrated with the recently emerged imaging approach of MPI was described in a paper by Bok *et al.* in 2022.^20^ This study was an important milestone because it showed that MENPs could provide a signal detectable with existing magnetic imaging modalities, thus paving a way to mapping the electric field activity deep in the brain. A high-efficacy implementation of using MENPs for wireless high-resolution neural recording was theoretically described in a paper by Zhang *et al.* in 2023.^21^ This study for the first time emphasized the importance of placing MENPs on the membrane. They showed that in this case the recorded signal could be increased by at least three orders of magnitude compared to the traditional scenario, when no focus is placed on a specific location of the nanoparticles in the cellular microenvironment.

Again, comprehensive experimental studies to demonstrate recording *in vitro*, *ex vivo* or *in vivo* remain to be conducted. However, based on the above theoretical studies, it could be concluded that MENPs for recording must be designed differently from MENPs for modulation. For example, one significant difference would be the average coercivity field of the nanoparticles; to maximize the sensitivity, this field must be significantly smaller for the recording mode compared to that for the modulation mode. Ideally, the coercivity should be smaller than the nanoparticle's field induced during neural firing. Hence, a focus must be made to significantly improve the control of the nanoparticle's size uniformity and crystallinity. The crystallinity quality is key to control the core's magnetic anisotropy, both magneto-crystalline volume and surface contributions, as well as provide adequate lattice matching between the core and the shell, while the size determines the non-volatility time of the magnetic core, as described above (eqn (4)). On one hand, to avoid the superparamagnetic state, the non-volatility time needs to be longer than the measurement time. On the other hand, to provide real-time recording, the non-volatility time needs to be shorter than the characteristic relaxation time of neural firing events under study. In other words, the size is a major factor to control this important trade-off.

It can be noted that the requirements on size and other properties significantly differ between modulation and recording processes. For example, during the recording process, it is important to ensure that the nanoparticles’ dipole moment variations never reach a threshold value required to induce local firing, thus avoiding any potential disturbance of the measured neural state. In contrast, during the modulation process, the induced electric dipole moment during magnetic field application is expected to exceed the local threshold value, thus causing the target modulation. Hence, the optimal solution could be to design different MENPs for modulation and recording. As described above, the nanoparticles’ properties could be properly controlled through the chemical composition to meet all the requirements. Arguably, integration of low-coercivity MENPs with the MPI imaging mode, while keeping the highest possible ME effect, would be the most viable route to map local electric field due to neural activity deep in the brain.

### Diagnostics: MENPs can function as MRI contrast enhancement agents and more

In addition to using MENPs to read cellular activity *via* their magnetoelectric effect, there is evidence to suggest that they could be used as a *T*_2_ weighted MRI contrast agent due to the ferrimagnetic properties of a magnetostrictive core like cobalt ferrite.^77,107^ In 2019, the first experiment to use MENPs as a MRI contrast enhancement agent in an *in vivo* experiment on non-human primates was described in a paper by Kaushik *et al.*^77^

The *T*_2_ contrast mechanism in MRI is predicated on the manipulation of transverse relaxation processes of proton spins within tissues. *T*_2_ contrast agents function by inducing localized magnetic field inhomogeneities in their immediate vicinity. These microscopic field distortions engender differential magnetic environments for proximal protons, thereby accelerating the dephasing of their spins. This enhanced spin–spin relaxation manifests as a reduction in *T*_2_ relaxation times, culminating in hypointense regions on *T*_2_-weighted images.

The efficacy of *T*_2_ contrast agents is largely attributable to their magnetic susceptibility effects. When subjected to an external magnetic field, these agents undergo magnetization changes, generating localized magnetic fields. These induced fields interact with nearby proton spins *via* dipole–dipole interactions, further exacerbating the dephasing process. While there already exist a number of *T*_2_ contrast agents, MENPs have shown some unique potential benefits. Previous studies have demonstrated the ability for MENPs to accumulate in tumor tissue, likely through a combination of the EPR effect and the ability to guide the nanoparticles with a magnetic gradient. Furthermore, because of the ME effect, unlike the conventional magnetic nanoparticles used as MRI contrast enhancement agents such as gadolinium nanoparticles, MENPs respond to local electric fields in a biological system under imaging, *e.g.*, at the cellular membrane. It is known that these fields can be relatively significant, *e.g.*, on the order of 100 000 V cm^−1^, across the dielectric membranes.^21^ Furthermore, these local electric fields can significantly differ between different cell types and sub-types,^108–110^ thus enabling a new cellular specificity detection mechanism. For example, this mechanism may prove useful in a contrast agent as it would functionally highlight tumor tissue.

### The first theranostic application study

According to the above analysis, the magnetoelectricity of MENPs should naturally allow for a truly theranostic treatment whereby the MENPs simultaneously function as MRI contrast agents, while being activated by the MRI field and specifically ablating the tumor tissue. In 2024, the first *in vivo* experiment where they used this theranostic capability of MENPs to treat a pancreatic cancer in a mouse model was described in a paper by Bryant *et al.*^107^ In their study, the *in vivo* efficacy was assessed in murine pancreatic ductal adenocarcinoma (PDAC) models (over 40 mice), comparing tumor volume reduction and 
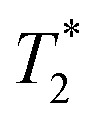
 relaxation time modulation across multiple doses (60 μg, 300 μg, and 600 μg). The 7-T MRI-activated MENPs at 300 μg and 600 μg induced significant tumor shrinkage, with complete responses observed in 33+% of mice receiving activated MENPs. Furthermore, the dose-dependent decreases in the MRI signal measured as 
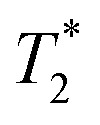
 relaxation times were strongly correlated with tumor reduction (*ρ* = −0.73, *P* < 0.001). Additional *in vitro* flow cytometry measurements indicated that MENPs induced apoptosis as the predominant mechanism of cell death, with minimal necrosis, ensuring precise and controlled cytotoxicity. Time-course analyses showed a progressive increase in apoptotic cell death over three hours post-activation. Histopathological evaluations revealed no significant toxicities in treated cohorts, indicating a favorable safety profile. This work is a significant milestone that directly proves the potential of MENPs to be used as a powerful theranostic agent to treat currently untreatable cancers.

### Other emerging configurations of MENPs

Furthermore, it is noteworthy that although the entire paradigm to leverage magnetoelectricity as a way to use magnetic fields to control local electric-field energy, that underlies fundamental biological processes, was launched using core–shell MENPs of the currently best studied composition,^1,3,8,12,43^ it is likely that novel compositions of MENPs or even other magnetoelectric configurations, potentially biodegradable, will emerge in the future, depending on a particular application. Such applications have already started to emerge.^32,85,111^ In 2022, a study in which they used a millimeter size magnetoelectric device to stimulate peripheral nerves was described in a paper by Chen *et al.*^32^ In 2024, a study where they created a biodegradable implant in the form of a flexible paper with embedded MENPs was described in a paper by Choe *et al.*^85^

## Conclusions

With the undeniable surge of both theoretical and experimental studies in recent years, the emergence of MENPs-based biomedical applications in the near future is likely inevitable. Due to their magnetoelectricity, MENPs present a powerful platform to tap (at the molecular level) into the complex electric-field-driven energy underlying fundamental biological processes. Furthermore, due to the reciprocal nature of the ME effect, MENPs naturally provide a two-way wireless connection with biological cellular networks, in turn enabling a dynasty of theranostic technologies. Arguably, this capability to simultaneously provide diagnostic and therapy is one of the most important properties of MENPs. In addition, due to this two-way wireless molecular-level communication capability, MENPs could become an enabler of the currently theoretical concept of the Internet of Medical Things (IoMT).^112^ The IoMT concept promises to bring the artificial intelligence (AI) driven healthcare to the next level.

According to the above analysis, different spatiotemporal patterns, *i.e.*, composed of one or a combination of specific frequency harmonics, with amplitudes matched to certain thresholds in neural circuits, could be used for distinguishing therapeutic and diagnostic properties of the nanoparticles. Nevertheless, despite the significant potential impact, this area of research/development is at a very early stage. Moreover, the developments of the two main theranostic functions, *i.e.*, therapeutic and diagnostic operations, respectively, have not progressed at equal rates. To date, according to this review, most biomedical applications have leaned towards therapeutic functions, arguably, because these functions are more straightforward to implement. Indeed, there have been a few dozens of papers published on therapeutic effects, while only a few papers on diagnostic effects. For comparison, the therapeutic application studies have included comprehensive experiments in *in vitro*, *ex vivo* and *in vivo* settings, with the *in vivo* studies conducted on numerous animal models, including rodents and NHPs. In contrast, the available diagnostic application studies have mostly been conducted at a theoretical level, only with one paper describing in an vitro experiment^106^ and one paper describing an *in vivo* study.^77^ Finally, there have been only one, *in vivo*, study where they combined the two functions.^107^ Such an uneven progress is not surprising. It is not trivial to wirelessly detect relatively small magnetic fields due to the ME effect of these nanoparticles in response to a local cellular activity. Arguably, the main reason might be the fact that the nanoparticles have not been properly utilized in most experiments. The interface between the nanoparticles and the cell membrane would need to be significantly improved by developing MENPs of more suitable, arguably, rectangular-flat-prism shapes and/or, alternatively, conjugating the nanoparticles with membrane-targeting bioreagents. Furthermore, the nanoparticles operate in significantly different regions of their *M*–*H* loops when used for therapeutic and diagnostic purposes. For therapeutic purposes, it is important to apply a magnetic field with a strength comparable to the anisotropy field. For the best studied composition of CoFe_2_O_4_@BaTiO_3_, such a field would be on the order of 1 T, which is relatively strong and not easy to control. In contrast, according to independent theoretical predictions,^19–21^ the typical magnetic field strength for diagnostic purposes needs to be orders of magnitude smaller to avoid any reversible or irreversible cell manipulation. This would lead to a significantly weaker field emanating from the nanoparticles during any theranostic process. In turn, this condition places a stricter requirement on the detection sensitivity used in the theranostic process, also limiting the spatial resolution. Hence, novel compositions and shapes of MENPs will likely emerge in the future. These novel compositions and shapes would be tailored to meet specific requirements on both therapeutic and diagnostic characteristics. Nevertheless, the above described first *in vivo* demonstration of a theranostic application proves that the future surge of such applications is inevitable.

Based on a physics-based analysis of the literature, this paper also identified some of the most eminent engineering challenges, mostly related to the field-controlled interaction between MENPs and cellular microenvironment, that need to be addressed prior to successful development of biomedical applications of MENPs. These challenges are equally important for both therapeutic and diagnostic applications. Several biomedical applications have been reviewed and analyzed from this perspective in more detail, also summarized in Table 1. To fully realize the potential of MENPs for these and other biomedical applications, future research must focus on refining their physical properties, including improving magnetic and electric field responses by ensuring perfect interaction with the cellular microenvironment. The authors hope that leveraging the insights shared in this analysis could help enhance the performance of MENPs in their future theranostic applications.

**Table 1:** Summary of MENPs’ biomedical applications and specific requirements, including relevant citations

Biomedical application	Requirement type	Specific details	Relevant citations
Neural modulation (therapy)	Localization on neural membrane	Surface functionalization or rectangular particles.	28,29,33,44,87,89,90
	A moderately high electric field needs to be applied (<0.01 mV nm^−1^)	Application of magnetic fields on the order of 1 kOe using CoFe_2_O_4_@BaTiO_3_ core–shell nanoparticles.	13,28–31,36,44,46,85,87,113
	Magnetic properties	Remnant magnetic moment below 10 emu per cc.	10,28,44,114
	Core–shell MENPs could be synthesized to respond to smaller magnetic fields (≪1 kOe)	NiFe_2_O_4_@BaTiO_3_ composition. For comparison, most described MENPs respond to fields above 1 kOe.	11,85,92
Cell electroporation (therapy)	Field threshold	Reversible electroporation: ≪1000 V cm^−1^	15,16,53,79,93,98
		Irreversible electroporation: >1000 V cm^−1^	43,94,98,99 and 107
	Localization	MENP needs to be directly on the membrane.	15,43
	Magnetic Properties	MENP should have the highest possible ME effect (≳1 V cm^−1^ Oe^−1^)	10,53
Targeted delivery (therapy)	Magnetic moment (if using a permanent magnet for localization)	MENPs should possess a relatively strong magnetic moment (>10 emu per cc).	43,53,107 and 115
	Surface charge	MENPs should have a negative surface charge.	43,53,116,117
	Apply AC fields of low strength and low frequency	For delivery across the membrane.	15,43,76,101,103–105
	Drug retention	Perfect drug retention until the field is applied, to avoid off target effects.	3,43,103,105,115
Recording, imaging (diagnostic)	Localization	MENPs should be on the membrane	21,53
	Magnetic properties	Enhance size uniformity to lower coercivity for MPI-based brain activity mapping.	19–21,106
	Safety	The dipole variations of the MENPs should never reach the threshold necessary to induce firing.	21,53
	ME effect	MENPs should have the highest ME effect possible.	10,21,106

## Data availability

Authors commit to provide all the associated data upon request.

## Conflicts of interest

Ping Liang and Sakhrat Khizroev are co-founders and shareholders of Cellular Nanomed, Inc., which is developing technologies and holds patents on magnetoelectric nanoparticles (MENPs) and their applications in medicine, including cancer treatment, brain–computer interface, brain stimulation, medical devices, and imaging.
